# Defoliation reduces soil biota – and modifies stimulating effects of elevated CO_2_

**DOI:** 10.1002/ece3.1739

**Published:** 2015-10-08

**Authors:** Marie Dam, Søren Christensen

**Affiliations:** ^1^ Terrestrial Ecology SectionDepartment of BiologyUniversity of CopenhagenCopenhagenDenmark; ^2^ Zealand Institute of Business and TechnologyBredahlsgade 1DK‐4200SlagelseDenmark

**Keywords:** Deschampsia, global change, grass, growing season, microbial biomass, microbial loop, nematodes, nutrient, rhizodeposition, soil

## Abstract

To understand the responses to external disturbance such as defoliation and possible feedback mechanisms at global change in terrestrial ecosystems, it is necessary to examine the extent and nature of effects on aboveground–belowground interactions. We studied a temperate heathland system subjected to experimental climate and atmospheric factors based on prognoses for year 2075 and further exposed to defoliation. By defoliating plants, we were able to study how global change modifies the interactions of the plant–soil system. Shoot production, root biomass, microbial biomass, and nematode abundance were assessed in the rhizosphere of manually defoliated patches of *Deschampsia flexuosa* in June in a full‐factorial FACE experiment with the treatments: increased atmospheric CO_2_, increased nighttime temperatures, summer droughts, and all of their combinations. We found a negative effect of defoliation on microbial biomass that was not apparently affected by global change. The negative effect of defoliation cascades through to soil nematodes as dependent on CO_2_ and drought. At ambient CO_2_, drought and defoliation each reduced nematodes. In contrast, at elevated CO_2_, a combination of drought and defoliation was needed to reduce nematodes. We found positive effects of CO_2_ on root density and microbial biomass. Defoliation affected soil biota negatively, whereas elevated CO_2_ stimulated the plant–soil system. This effect seen in June is contrasted by the effects seen in September at the same site. Late season defoliation increased activity and biomass of soil biota and more so at elevated CO_2_. Based on soil biota responses, plants defoliated in active growth therefore conserve resources, whereas defoliation after termination of growth results in release of resources. This result challenges the idea that plants via exudation of organic carbon stimulate their rhizosphere biota when in apparent need of nutrients for growth.

## Introduction

Soil biota plays a significant role in biogeochemical cycling and their responses to global change are therefore considered important at the ecosystem scale (Brussaard 1998; Bradford et al. 2002), but are remarkably understudied (West et al. 2006; Bardgett et al. 2013). The interactions between the aboveground and the belowground spheres are complex relationships affected by biotic as well as abiotic factors.

Defoliation is a disturbance of the plant–soil system by partial removal of the aboveground biomass. Defoliation effects belowground depend on abiotic factors such as climate as well as biotic factors such as plant growth phase (Guitian and Bardgett 2000; Wilsey 2001; Yeates et al. 2003; Ilmarinen et al. 2005; Lau and Tiffin 2009; Yeates and Newton 2009; Stevnbak et al. 2012). Defoliation effects on the plant–soil interactions may relate to whether plants stimulate decomposition through the exudation of low‐molecular‐mass carbon compounds when in apparent need of nutrients (Griffiths and Robinson 1992). The exudates would then feed the microbial loop and increase the microbial grazers and higher trophic levels of the soil food web (Bonkowski et al. 2000). There have been several studies with both negative (Holland and Detling 1990; Northup et al. 1999; Nguyen and Henry 2002) and positive (Holland et al. 1996; Hamilton and Frank 2001) effects on carbon release from roots upon defoliation. Similarly, defoliation had different effects on amount of microorganisms going from a decrease in microbial biomass (Guitian and Bardgett 2000; Williamson and Wardle 2007) to an increase in number of bacteria (Mawdsley and Bardgett 1997), but this did not result in an increased microbial activity.

Defoliation effects on soil biota will depend greatly on which plant responses are affected by the action. Litter quality may decrease at elevated CO_2_ due to reduced nitrogen content and therefore slower decomposition (Ball 1997). Water use efficiency could increase at elevated CO_2_ potentially increasing soil moisture followed by increased decomposition and nutrient mineralization (Field et al. 1995). Finally, root biomass depends strongly on availability of resources for plants, and rhizodeposition is important for the rhizosphere biota (Jones et al. 2009). Defoliation effects have been observed on resource allocation within the plant and in the rhizosphere. In grassland sampled in the middle of the growing season, defoliation by grazing resulted in increased aboveground production (Frank 1998) and an increased transport of N and P from roots to shoots (Mikola et al. 2009). Mikola et al. (2009) found no stimulation of either mineralization or soil fauna by defoliation of plants in active growth**.** Similar results were obtained in microcosms with newly established grass in active growth: At field nutrient levels, defoliation altered allocation of C and N, but did not stimulate either microbial activity or abundance of microbial grazers (Ilmarinen et al. 2008). In another microcosm experiment with defoliation of grasses in different phases of growth, Ilmarinen et al. (2005) suggest that the reduced root C concentration they find in defoliated plants could be due to increased C allocation to growing shoots at the expense of roots following defoliation, as found in Caldwell et al. (1981), Briske et al. (1996), and Strauss and Agrawal (1999). However, they also find that plants defoliated in the later stages of the growing season increased the root mass relative to plant mass.

Stimulating effects of defoliation on soil biota have been reported, but often in studies performed under less favorable conditions for plant growth, after the most productive part of the growing season. Defoliation in a cool Scottish upland in September (Ostle et al. 2007) or in water‐limited grassland of Yellowstone in the driest month of July (Hamilton et al. 2008) both resulted in transfer of more photosynthate to soil biota. In a Danish temperate heathland at the end of the growing season in September, defoliation resulted in increased carbon flow through the soil biota and more so at elevated CO_2_ (Stevnbak et al. 2012). Based on the above‐mentioned studies, it seems as if defoliation of actively growing grass does not induce carbon release from plants to soil biota, whereas carbon exudation may increase when the active growth phase is over.

Elevated CO_2_ generally results in an increase of abundance and activity at the bottom of the food web, that is, of bacteria, fungi, and microfauna (protozoa and nematodes) as found in a meta‐analysis of soil biota response to global change (Blankinship et al. 2011). Now, elevated CO_2_ will not occur alone but in combination with climatic changes such as elevated temperature and altered precipitation pattern. Responses of soil biota to global change are unique for each global change factor with positive effects of elevated CO_2_ and precipitation and negative effects of warming (Blankinship et al. 2011). Interactions between different global change factors may create responses not predicted by single‐factor experiments, for example, elevated CO_2_ increased net primary production in a grassland but in combination with elevated precipitation, temperature, or both, elevated CO_2_ had a negative effect on primary production (Shaw et al. 2002). The interaction between elevated CO_2_ and temperature has been modeled with three different biogeochemical models (Norby and Luo 2004) with different results. This shows that multifactor experiments are needed to increase our understanding of the processes. One of the few recordings of multiglobal change factors with impact on soil biota revealed significant effects involving elevated CO_2_, N deposition, and summer drought (Eisenhauer et al. 2012). Here, CO_2_ was the global change factor affecting most soil biota groups, with increasing abundances at micro‐, meso‐, and macrofauna level. Furthermore, CO_2_ turned out to be the only global change variable playing a role when building a SEM model of global change effects on the soil food web (Eisenhauer et al. 2012). The likely explanation as already stated by Ostle et al. (2007) is that environmental changes affecting the quantity and quality of photosynthate‐C inputs to the soil impact the biology that regulates the soil C cycle.

In this study, we defoliated grass in active growth. The study was performed in a field site in a multifactor FACE experiment where CO_2_, temperature, and precipitation are manipulated to simulate predicted global change (IPCC 2013). This allowed us to test how this disturbance affected aboveground–belowground interactions under influence of elevated CO_2_ as well as predicted climatic changes. If defoliation causes plants to actively increase rhizodeposition in order to gain nutrients from soil biota activity, we would expect a stimulation of soil biota at defoliation and more so at elevated CO_2_ where nutrient availability in soil is reduced.

## Materials and Methods

### Site description

The experiment took place at the CLIMAITE experimental site (55°53′ N, 11°58′ E) – a FACE facility approximately 50 km northwest of Copenhagen, Denmark. The site is a dry, temperate heathland, dominated by the dwarf shrub *Calluna vulgaris* (L.) and the perennial grass *Deschampsia flexuosa* (L.). The soil is a well‐drained, nutrient‐poor sandy deposit with a pH of 4–5 and an organic top layer ranging from 2 to 5 cm in depth. Long‐term annual mean air temperature is 8.0°C, and annual mean precipitation is 607 mm (Danish Meteorological Institute).

### Experimental design

The setup consists of twelve 7 m diameter octagons. Each octagon is divided into four plots receiving either (1) summer drought (D) by automatic rainout shelters; (2) passive nighttime warming (T) of air and soil by reflectance curtains 50 cm above ground; (3) a combination of drought and warming (TD); or (4) neither drought nor temperature treatment. Furthermore, six of the twelve octagons are under ambient (A) atmospheric CO_2_ concentrations and the other six subjected to an elevated (CO_2_) CO_2_ concentration (510 ppm in a free air CO_2_ enrichment setup, FACE). The experiment thus has a full‐factorial design arranged in blocks of pairwise octagons representing all combinations of D, T, and CO_2_, including an untreated control for reference (A). Hence, eight treatments with six replicates, in total 48 plots, arranged in a split plot design (Mikkelsen et al. 2008). The warming treatment elevates the air and soil temperature by 1–2°C. The drought continues for 2–5 weeks or until soil water content falls below 5 vol.% water content in the top 20 cm of the soil (Mikkelsen et al. 2008). The drought effect lasts into the fall, but by October, the soil moisture is only 1% lower in the drought‐treated plots (Dam et al. in prep.). Nitrate in lysimeter water in the upper 15 cm mineral soil was reduced between ambient and elevated CO_2_ (*P* = 0.031) from 2.3 to 1.1 ppm N (K. S. Larsen et al., unpubl.). The experimental area is protected from large herbivores by fencing.

### Defoliation treatment

The entire climate manipulation design in operation for 6 years was overlain with a +/− defoliation treatment on areas with *Deschampsia flexuosa*. In each plot, two circular units of 0.07 m^2^ were marked off in segments where *D. flexuosa* was dominant. Two of the plots did not have a sufficient area of grass leaving us with 46 plots (six treatments with six replicates, two treatments with five replicates, *n* = 92). The vegetation in the grass units was either left nondefoliated as a control or defoliated by cutting. Cutting was performed manually four times, every 6–8 days starting June 1st after the annual drought treatment had removed precipitation throughout May. The cuttings were removed from the plots. Before the first cutting, the grass height of the units was assessed. The average of all units was 14.4 cm ±3.9 with no treatment differences. At each defoliation event, the vegetation was cut down by 1/6 of the pretreatment median height in each individual defoliation unit to simulate the effect of foliar insect herbivores such as grasshoppers (e.g., Avanesyan and Culley 2015). Thus, by the end of the treatment, the defoliation had removed 2/3 of the original vegetation, and the median height was approximately 8–10 cm above the soil, depending on the original median height.

### Soil sampling

At June 27th, soil samples were randomly collected in all 92 units by coring. One larger core (4 cm diameter, 15 cm deep) was sampled for root biomass determination. Three cores (2 cm diameter, 8 cm deep) were retrieved and mixed to cover spatial variability. The soil from the 2 cm cores was analyzed for soil moisture content, SOM, nematode numbers, and microbial biomass by chloroform fumigation. Root C:N, substrate‐induced respiration (SIR), and protozoan numbers were estimated, too, but these data showed no significant response and are not presented here. The soil samples were transported in coolers and kept at 5°C until processed. To deal with the large number of samples, they were processed over 8 days, with the different treatments distributed evenly between the days to avoid bias. This staggered processing is furthermore accounted for in the statistical model. When processed, aboveground plant biomass was removed and the rhizosphere carefully cut up for homogenization.

### Shoot and root analyses

The grass height of all units (+/− defoliation) was assessed at each defoliation treatment as well as at the end of the defoliation treatment. These values were used for estimation of shoot production. The roots from each 4 cm core were carefully washed over a 2‐mm sieve, and all root material was collected and dried at 80°C and weighed. From the mixed 2 cm cores, subsamples of 5 g soil were dried at 80°C for 48 h for soil moisture determination and combusted at 550°C for 6 h for SOM determination by loss on ignition.

### Soil microbial biomass and growth

A subsample of 10 g of soil mixture was fumigated in ethanol‐free chloroform (CHC13) for 24 h to release the nutrients in the soil microbial biomass (Jenkinson and Powlson 1976; Tate et al. 1988). After fumigation, the soil was extracted in 50 mL 0.5 mol/L K_2_SO_4_ for 1 h and filtered. Simultaneously, another subsample was extracted in the same manner but without fumigation to recover the soil inorganic nutrients. Due to problems with the fumigation procedure, only 1/3 of the data were analyzed. Total organic carbon (TOC) (fumigated samples) and dissolved organic carbon (DOC) (nonfumigated samples) were measured on Shimadzu TOC‐5000A total organic C analyzer using the infrared gas detector (IRGA) method. Microbial carbon was calculated using the extractability factor KEC = 0.45, to account for the microbial biomass C that is not released by fumigation and extracted by K_2_SO_4_ (Jonasson et al. 1996): Microbial C = (TOC – DOC)/KEC. Microbial growth was assayed as fractional increase in respiration rate (respiration rate 4–20 h/respiration rate 0–4 h, Scheu 1992) in agitated soil slurries amended with carbon (Wamberg et al. 2003).

### Soil fauna

Nematodes were extracted from 5 g (fresh weight) of soil by a modified combination of the Baermann pan and the Whitehead tray (Whitehead and Hemming 1965) extraction methods. Samples were extracted for 48 h, and nematodes were then counted at ×40 magnification using a dissecting microscope. After counting, the samples were fixed in a 4% formaldehyde solution. They were later analyzed for nematode community composition of trophic groups. Based on mouth part morphology, the nematodes were identified to one of five feeding groups (Yeates et al. 1993) under a dissecting microscope at ×40 magnification.

### Statistics

We analyzed effects of global change manipulations and the defoliation and all possible interactive effects on the plant–soil system: With the three climate change factors (CO_2_, temperature, and precipitation/ drought) as well as defoliation as fixed factors, we used mixed linear models to test the effect on every measured plant and soil variables. The statistical model was extended with a random statement to account for random variation introduced by the experimental design. As random factors, we used block (representing pairs of octagons including all treatments), CO_2_ nested within block, warming nested within CO_2_ and block, and drought nested within CO_2_ and block. Due to lack of replicates for microbial biomass, we have CO_2_ and defoliation as fixed factors for this parameter and CO_2_ nested within block as random statement. We applied log transformation when necessary to obtain normality. All data were analyzed in R (R Development Core Team, 2013) using the lmer function from the lme4 package (Bates et al. 2014). The anova function from the LmerTest package was used to obtain *P*‐values. Models were reduced based on evaluation of *F* values using the step function (LMERConvenienceFunctions). In the results reported below, only the factors kept in the model after reduction are shown for each analysis.

## Results

**Figure 1:**
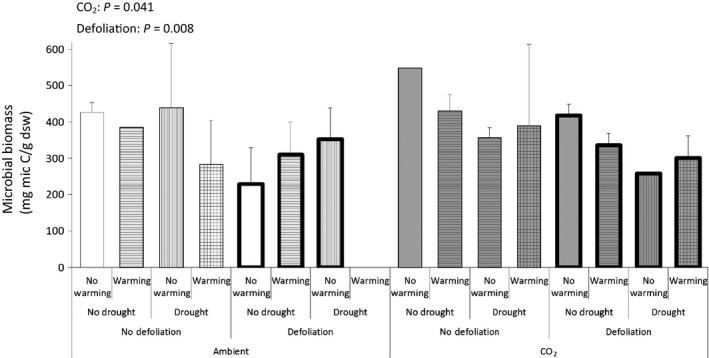
Microbial biomass. Means with SE bars (*n* = 1–3). Lack of replication (see Materials and Methods) only allowed us to run a two‐factor mixed linear model (CO_2_ * defoliation) on these data. Significant effects at *P* < 0.05 are displayed.

**Figure 2:**
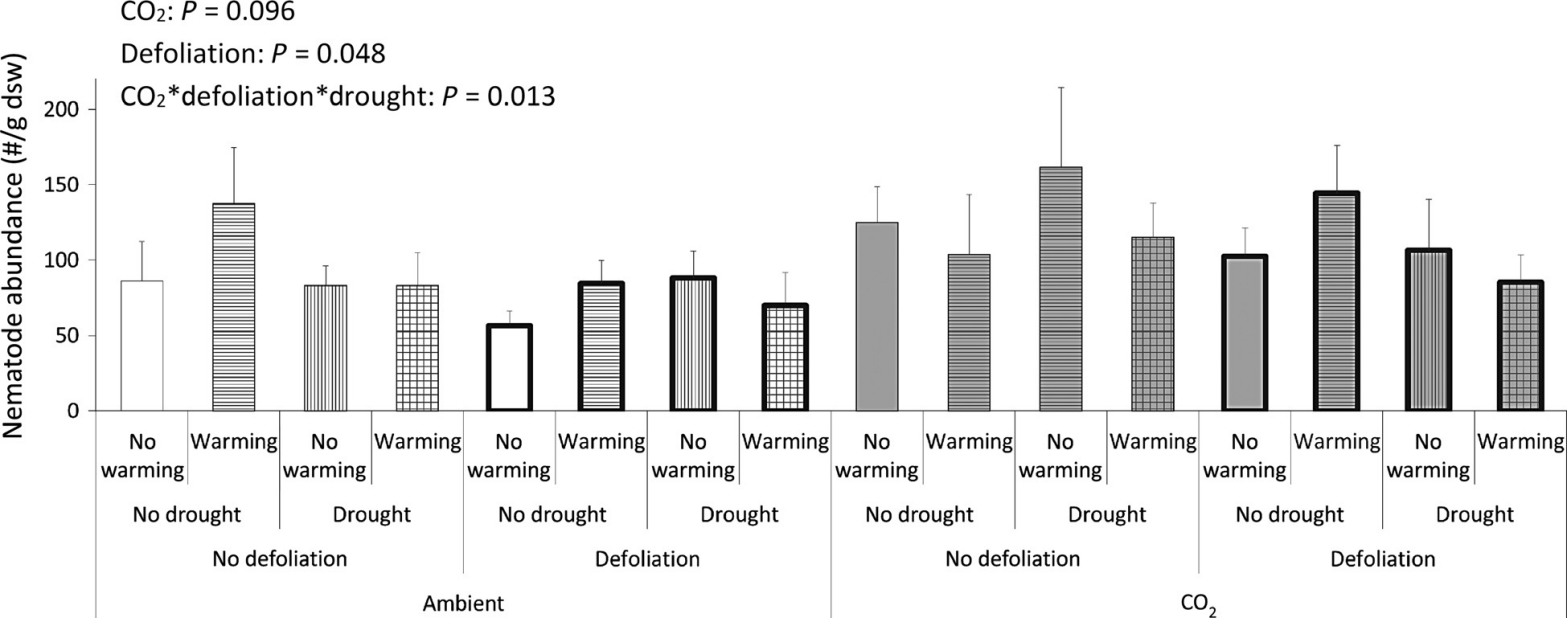
Nematode abundance. Means with SE bars (*n* = 6). Effects at *P* < 0.1 are displayed.

**Figure 3:**
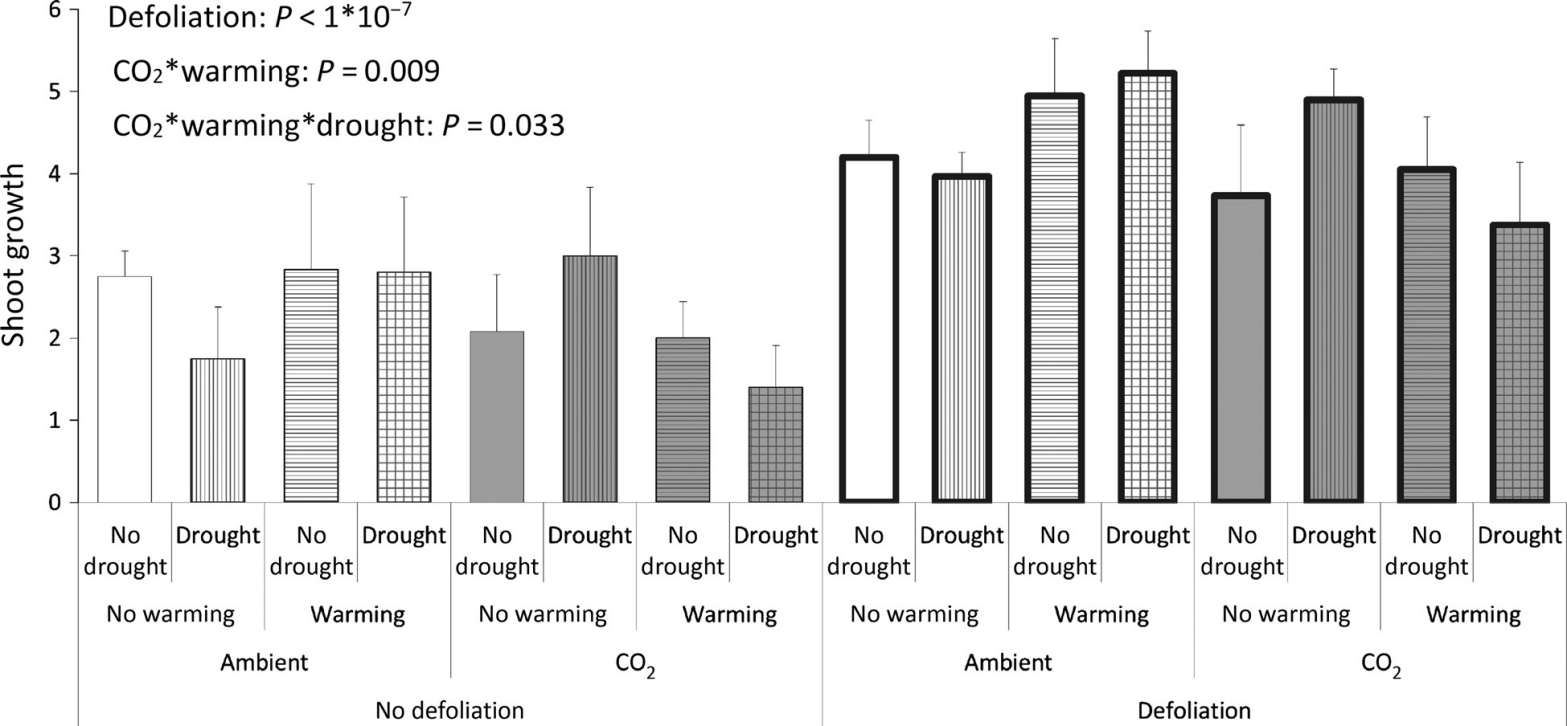
Shoot productivity: Growth of *Deschampsia flexuosa* in treatment units since first defoliation date. For defoliation‐treated units, the cuttings are included in cumulative values for growth. Values are means with SE bars (*n* = 6). Significant effects at *P* < 0.05 are displayed.

We found a negative effect of defoliation on soil biota exerted via aboveground–belowground interactions. The model showed statistically significant main effects of defoliation on microbial biomass (Fig. 1) and on nematode abundance (Fig. 2). At the same time, there is a considerable regrowth of the defoliated *D. flexuosa* (Fig. 3) – comparably larger than the growth of the nondefoliated plants in the same time span. The results also show that the plants were indeed in active growth when defoliated, as there is a considerable growth of the nondefoliated plots, too (Fig. 3). The defoliation was not just numerically but also statistically the most significant effect on shoot growth. Warming reduces shoot growth and drought increases this effect, both at elevated CO_2_. These interactions between global change treatments on shoot growth were numerically smaller and statistically less strong compared to defoliation (Fig. 3).

For nematode abundance, the interaction between, defoliation, drought, and CO_2_ treatment was significant (Fig. 2). Drought and defoliation each reduces nematode abundance at ambient CO_2_, whereas only the combination of the two reduces nematode abundance at elevated CO_2_ (Fig. 2). The relative abundance of nematode feeding groups was not affected by the treatments. The average distribution was 45% bacterivores, 30% herbivores, 15% fungivores, and 5% omnivores and predators (5% were unidentified). Microbial biomass showed a numerically small, but statistically significant increase at elevated CO_2_ (Fig. 1).

**Figure 4:**
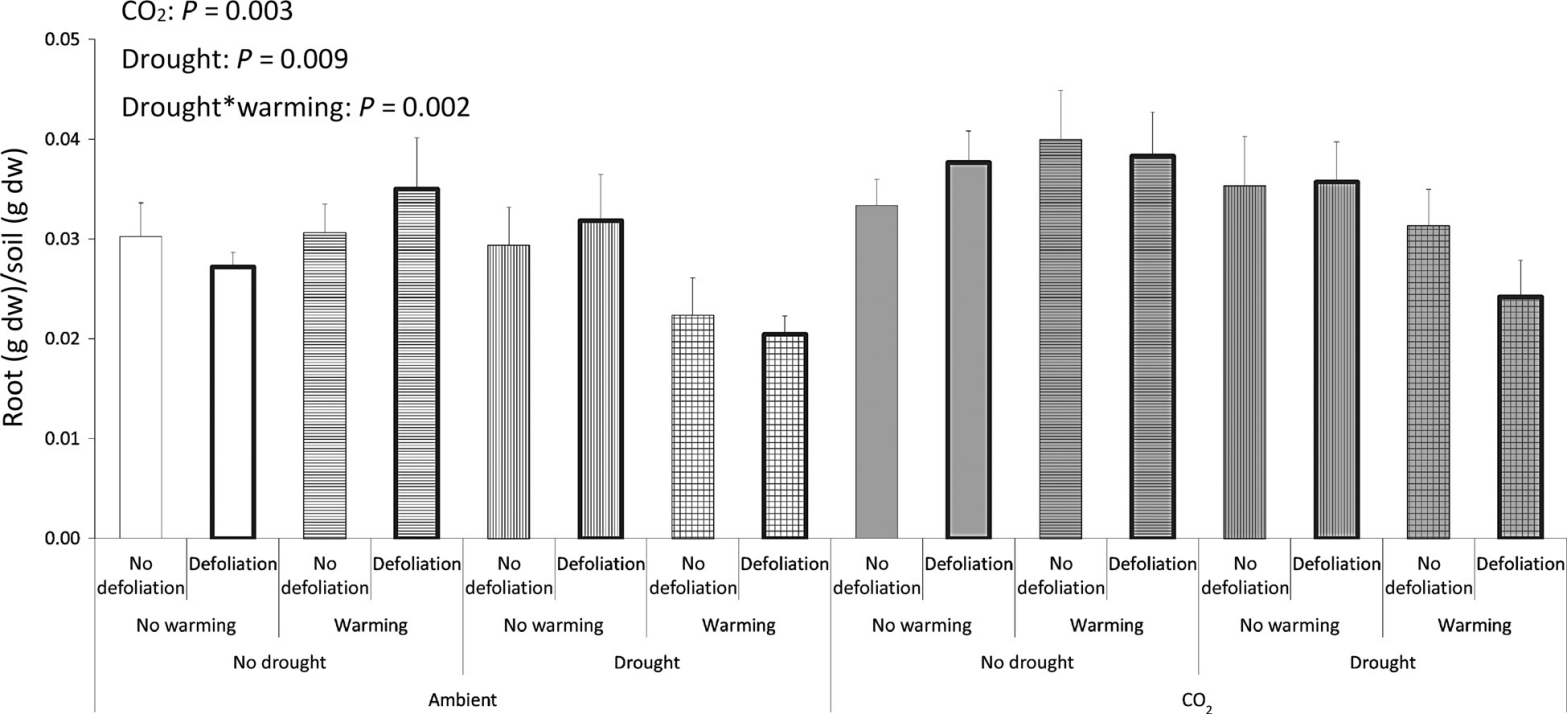
Root density. Means with SE bars (*n* = 6). Significant effects at *P* < 0.05 are displayed.

The elevated CO_2_ led to increases of two important links in the belowground food chain: The model showed positive statistically significant main effects of CO_2_ on root density (Fig. 4) and microbial biomass (Fig. 1). Drought and temperature had less pronounced effects on the system and primarily affected root density. Root density was reduced by drought and temperature enforced drought effects, further decreasing density (Fig. 4).

## Discussion

### Belowground response to defoliation depends on plant growth phase

The present study was performed in June, before seed‐set, where we expected that the plants were still investing a considerable amount of resources aboveground. In Stevnbak et al. (2012), a comparable amount of aboveground biomass of *Deschampsia flexuosa* was removed in the FACE experiment of the present study by grasshopper defoliation in September (after flowering and seed‐set, at the end of the growing season). Indeed, we saw a considerable regrowth of the grasses contrary to the September results from Stevnbak et al. (2012), where there was no compensatory growth in the defoliated grasses. However, contrary to Stevnbak et al. (2012), we found that aboveground defoliation reduced belowground biota – both nematode abundance and microbial biomass – and did not stimulate microbial growth (data not shown). The rhizosphere biota is presumably limited by easily available C as there are stimulating effects of elevated CO_2_, which through increased photosynthesis is likely to also increase C allocation belowground. Further, soil water nitrate was lower in the present study than in the study of Stevnbak et al. (2012) (K. S. Larsen et al., unpubl.), suggesting greater nutrient limitation in June than in September. As the defoliation does not have a stimulating effect, it seems reasonable to assume that the difference in the results of the two studies is at least in part caused by differences in allocation of resources belowground and the derived changes in root exudation, determined by growth phase and the need for resources aboveground for production of biomass, photosynthesis, and flowering/seed‐set. This difference in plant growth phase is confirmed by the lack of growth of nondefoliated vegetation during the September study, while the June study shows a considerable growth even in the nondefoliated units. In accordance with this, Frank (1998) finds a positive relationship between forage consumption and plant production in the growing season, and Wilsey (1996) finds an increased shoot production of grass defoliated soon after having been brought out of mimicked winter dormancy. Also in support of our results on investment of resources aboveground instead of in root exudation in plants defoliated before seed‐set, Ilmarinen et al. (2008) find a reduced allocation of C to roots and an increased allocation of N to shoots – without a corresponding increase in N uptake – upon defoliation. The study was performed on relatively young plants still in active growth and indicates an altered internal allocation of C and N in the plant rather than increased uptake and shows no stimulation of soil biota at defoliation (Ilmarinen et al. 2008). A study on defoliation of 8‐week‐old plants in microcosms (Stanton 1983) and a grassland field study of defoliation effects in spring (Todd 1996) showed reduced nematode abundances comparable to our findings. In line with this, an experiment where defoliation of grass was performed in both early and late growth phase resulted in a reduced microbial biomass early but increased microbial biomass in the late growing phase (Guitian and Bardgett 2000). Our results are obtained in a heathland with a grass cover of naturally low diversity. Under a more diverse plant cover, belowground effects might differ.

### CO_2_ increases soil biota and belowground plant biomass

Root mass and microbial biomass both increase at elevated CO_2_ in agreement with previous results from the experimental sites, observing increases in plant net photosynthesis at light saturation (Albert et al. 2011), biomass of roots (Arndal et al. 2014), and in soil respiration (Selsted et al. 2012). These components were all stimulated either by elevated CO_2_ alone or in interaction with drought or temperature. This is most likely due to the increased input of C into the belowground food chain from the increased CO_2_ available to aboveground photosynthesis. This result is in line with Eisenhauer et al. (2012) who found elevated CO_2_ to increase root and shoot biomass, and found the root biomass to be a determining factor for the soil food web. Hence, as hypothesized, we might see more organisms in the decomposer food web at future CO_2_ levels. The significant interaction between CO_2_ treatment, defoliation, and drought suggests that elevated CO_2_ creates more robust nematode populations, which it takes a combination of two stressors (drought and defoliation) to reduce. It takes only one stressor (drought or defoliation) to reduce nematode numbers under present day CO_2_.

In Stevnbak et al. (2012), the defoliation‐induced stimulation of belowground biota and nutrient availability is greater under elevated CO_2_ where photosynthetic capacity of grass plants is increased (Albert et al. 2011), where they grow more roots (Arndal et al. 2014) and thus contain more resources. In the present study, CO_2_ stimulated soil biota. Even when significantly reduced by defoliation and drought, the nematode abundance was numerically higher at elevated CO_2_ than under ambient CO_2_. Hence, in the two otherwise contrasting parts of the growing season, increased CO_2_ stimulates soil biota and thereby likely the decomposer capacity (Blankinship et al. 2011; Eisenhauer et al. 2012) partly due to increased rhizodeposition (Eisenhauer et al. 2012).

## Conclusion

It seems that the often proposed mechanism of plants feeding their belowground microbial loop when in immediate need of nutrients (Bardgett et al. 1998; Bonkowski 2004) is not present in this natural system, even though it is indeed relatively nitrogen limited and more so at elevated CO_2_ (Larsen et al. 2011). The present study shows that exudation and belowground allocation of resources to the advantage of the soil biota does not occur when the perennial plant is in need of resources for shoot growth.

We therefore propose that defoliation effect depends on plant growth phase: If the results from Stevnbak et al. (2012) and other studies showing a stimulation of belowground biota by defoliation (Mikola et al. 2001; Ostle et al. 2007; Hamilton et al. 2008) were indeed due to plants releasing carbon to feed the microbial loop when in need of nutrients, we would expect a more pronounced response when plants are in active growth than when the growth conditions are less favorable and the growing season is terminating. However, when we compare defoliation impact on soil biota before seed‐set (this study) with impact after seed‐set (Stevnbak et al. 2012), we find that the aboveground–belowground interactions upon defoliation seem to depend on prioritizing of resources related to aboveground growth rather than on the plants induction of the rhizosphere associated biota. These results emphasize the need for further investigation into whether plants are strategically regulating the life around its roots or if their inputs into the soil simply reflect differences in flow of resources depending on varying needs within the plant.

In the face of predicted increased CO_2_ levels in the atmosphere and the derived increased C input belowground and more abundant decomposer community demonstrated in this study, it therefore stands to reason to consider that management of grazing intensity of natural areas during the season could help modify the effects, as defoliation and CO_2_ worked antagonistically in the productive part of the season (June), whereas the effects were synergistic later in the season (September).

## Conflict of Interest

None declared.
